# Cohort Profile: Shiraz Pediatric Liver Cirrhosis Cohort (SPLCCS)

**DOI:** 10.34172/aim.2023.35

**Published:** 2023-04-01

**Authors:** Nasrin Motazedian, Bita Geramizadeh, Seyed Mohsen Dehghani, Negar Azarpira, Mahdokht Hossein Aghdaei, Ramin Yaghobi, Alireza Shamsaeefar, Kourosh Kazemi, Mohammad Hossein Karimi, Alireza Mirahmadizadeh, Amirali Mashhadiagha, Maryam Ataollahi, Homa Ilkhanipoor, Mitra Basiratnia, Hamid Nemati, Maryam Ekramzadeh, Anahita Sanaei Dashti, Saman Nikeghbalian, Seyed Ali Malekhosseini

**Affiliations:** ^1^Transplant Research Center, Shiraz University of Medical Sciences, Shiraz, Iran; ^2^Abu Ali Sina Organ Transplant Center, Shiraz University of Medical Sciences, Shiraz, Iran; ^3^Non-Communicable Diseases Research Center, Shiraz University of Medical Sciences, Shiraz, Iran; ^4^Student Research Committee, Shiraz University of Medical Sciences, Shiraz, Iran; ^5^Pediatric Department, Shiraz University of Medical Sciences, Shiraz, Iran; ^6^Department of Clinical Nutrition, School of Nutrition and Food Sciences, Shiraz University of Medical Sciences, Shiraz, Iran

**Keywords:** Adolescent, Child, Liver diseases, Liver transplantation, Longitudinal study, Mortality

## Abstract

Liver diseases in children and adolescents are a significant and arising public health issue and should be surveyed from different dimensions (clinical and para-clinical, psychological, socio-economic) and in diverse populations. Shiraz Liver Transplant Center, Shiraz, Iran is the only center for pediatric liver transplantation and its pre-operative evaluations. This provides a unique and valuable situation for studying this vulnerable population. The Shiraz Pediatric Liver Cirrhosis Cohort Study (SPLCCS) was established to assess cirrhotic children, the course of their disease, and treatment over time. This cohort study aimed to prospectively evaluate the natural course and factors that contributed to complications and death of children with chronic liver disease in the region. SPLCCS was launched in September 2018 after obtaining ethical approval; until August 2022, 370 children with end-stage liver disease were enrolled and followed every six months. Here, the cohort’s features, the included population’s baseline characteristics, and primary outcomes are reported.

## How Did the Study Come About?

 The worldwide prevalence of liver diseases has increased among children and adolescents, rising from 72 4200 in 1990 to 917 800 in 2017, with an annual increase of 0.13% during the mentioned time. The prevalence of obesity has resulted in non-alcoholic fatty liver disease in the pediatric population. Chronic liver disease significantly impacts public health issues with increased mortality and morbidity and decreased quality of life, leading to overwhelming costs without curative or supportive treatment. Chronic liver disease in children is the forerunner of adult chronic liver diseases and hepatocarcinoma. Thus, prevention and treatment in childhood can be cost-effective. Liver disease is usually undiagnosed or diagnosed late in children because of the non-specific clinical manifestations, such as easy fatigability, anorexia, abdominal pain, or itching sensation in the primary stages.^1-4^

 Cirrhosis can be the end point of many conditions affecting the liver in different age groups. Biliary atresia and genetic-metabolic diseases account for most cases in infancy, compared to autoimmune hepatitis, Wilson’s disease, and primary sclerosing cholangitis which are more prevalent in older children and adolescents.^5^ Liver diseases need strong attention for developing non-invasive tests to detect fibrosis in early stages and promptly treat and stop or slow the progression, as a growing public health concern.^6,7^ The prognosis in cirrhotic patients deeply relies on the underlying etiology, severity, association of complications, and comorbidities.^8^

## Why Was the Cohort Set Up?

 A cohort study is a standard prognostic investigation in which a group of people with a particular condition or set of characteristics are followed over a period of time.^9^ At the start of the study period, various factors influencing the outcomes are repeatedly measured. Evaluation of different dimensions (clinical, para-clinical, psychological, social, and economic) of this disease over time is essential before transplantation to allocate the patients for liver transplantation and also to increase survival and improve the quality of life.

 Shiraz Liver Transplant Center is the only center for pediatric liver transplantation in Iran. Children and adolescents with cirrhotic diseases who need to be evaluated for liver transplants are referred to this center from all over Iran. This provides us with a unique and valuable situation for studying this population. The Shiraz Pediatric Liver Cirrhosis Cohort Study (SPLCCS) was established to assess cirrhotic children, the course of their disease, and treatment over time. Moreover, the significant sample size and regular follow-up of patients provide excellent settings for longitudinal studies.

 This study aimed to prospectively evaluate the natural course, and factors that contributed to complications and death of children with chronic liver disease who were referred to Shiraz Liver Transplant Center. This survey among this vulnerable population can lead to progress in this field. Taking interventional and preventive measures, setting up a network encouraging research and advancements in pediatric hepatology, and optimizing and standardizing the management and treatment of children with chronic liver disease are among the potential objectives that can be reached.

 SPLCCS was launched in September 2018 after approval of its protocol by the ethics committee of Shiraz University of Medical Sciences. Written informed consent was obtained from participants’ parents or legal guardians. This is an open cohort study, and enrollment is open up to now.

## What Does the Study Cover?

 The primary aims of the SPLCCS were:

- Identifying the demographic features, underlying liver diseases, frequency of comorbidities, malnutrition, growth failure, sexual health status, and delayed puberty,
- Establishing a bio-specimen bank for blood, hair, and nail samples to be used in basic sciences studies with a cross-sectional or nested case-control design,
- Creating a suitable and reliable source for extensive clinical research (interventional studies),
- Providing reliable evidence for the healthcare stakeholders and policymakers,
- Linking clinical research and basic sciences through patients’ clinical information and biobanks,
- Evaluating nutritional and medical interventions inchildren with liver diseases.

## Who Is in the SPLCCS?

**Figure 1:**
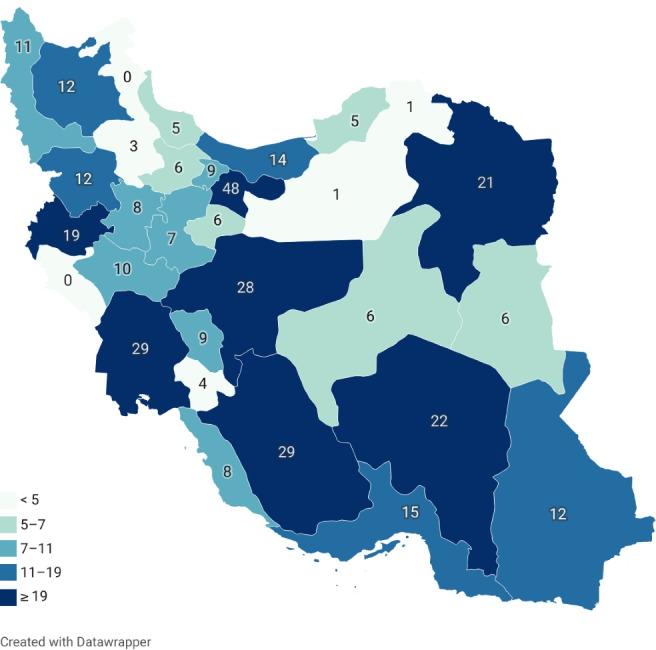
Distribution of Participants Enrolled in SPLCCS from Different Provinces of Iran.

 This study was performed on children under 18 years of age with chronic liver disease who were referred to Shiraz Transplant Research Center by pediatric gastroenterologists. Since Shiraz Liver Transplant Center is the only center for pediatric liver transplants in Iran, the participants came from different parts of the country; however, the center is located in Shiraz metropolitan area which is the capital of the Fars province. The distribution of participants is demonstrated in Figure 1. All of the children were invited, visited, and examined by pediatric gastroenterologists. Diagnosis of liver cirrhosis is based on clinical manifestations, laboratory and radiographic investigations, and finally, liver biopsy establishes the final diagnosis. However, it is not necessary in most cases. The only exclusion criterion was unwillingness to participate for any reason and at any stage of the study. With comprehensive explanation of the study and its future benefits by the pediatric gastroenterologist, all of the invitees accepted the enrollment. Each subject was interviewed by the trained staff of the cohort clinic. Before the interview, written informed consent was obtained from each participant or her/his parents or legal guardian.

 After interviewing based on pre-designed questionnaires and physical examinations, samples of blood (10 milliliters), hair (3 centimeters from the base of the scalp), and nails (trimmings gathered from ten toenails) were collected by a trained technician. All blood samples were immediately processed in the laboratory of Shiraz Transplant Research Center. Blood samples were kept in refrigerators ( + 4 ˚C) before transferring to the laboratory; the maximum duration between blood collection and final processing was eight hours. The plasma was collected, aliquoted into 1 mL portions, and stored frozen at -80 ˚C. Then, the buffy coat was collected from the rest of the blood samples, aliquoted in portions of 500 µL, and stored at -80˚Celsius. The hair and nail samples were stored at room temperature at Shiraz Transplant Research Center.

## How Were the Subjects Being Followed Up?

 Participants were followed up biannually after enrollment time. The databases of Shiraz Organ Transplant Center were also reviewed every six months to look for transplantation or mortality of the study subjects. A minimum of five years of follow-up was expected in this cohort study. In the cohort, 98% of participants had a private telephone line. Contact information including parental mobile phone numbers as well as two others from family members, neighbors, or close friends were taken at the time of registry.

 The cohort staff filled a follow-up pre-designed questionnaire with a phone call and recorded the patient’s status (alive, dead, transplant, hospitalization) and completed a pre-defined review of systems including musculoskeletal, nervous, endocrine, cardiovascular, lymphatic, respiratory, digestive, urinary, and the reproductive system. Then, they asked the parents to send the latest laboratory data (complete blood count, liver function tests, renal function tests, and routine coagulation tests) and vaccination data. Until August 2022, the SPLCCS team enrolled 370 children with end-stage liver disease. A total of 283 participants were followed once, 250 participants twice, 173 participants three times, and 103 participants four times. By now, ten (2.7%) cases have been lost to follow-up. The SPLCCS team recorded three types of outcomes: death (from any cause), liver transplantation, and hospitalization for any reason. The parents were asked to provide detailed information about death and hospital admission (place, date, and cause). Liver transplantation information was provided by the Shiraz Organ Transplant Center.

## What Was Found in SPLCCS?

 We analyzed the data on the baseline demographic characteristics, outcomes, and causes of mortality among children. The data was extracted fromRegistry of Pediatric Liver Cirrhosis (IR.SUMS.REC.1399.530). The baseline demographic and clinical characteristics of the cohort participants are listed in Table 1.

**Table 1:** Demographic and Clinical Features of the Study Participants

**Variables**			**Total; n=370 (%)**
Gender		Male	182 (49.2)
		Female	188 (50.8)
Age, years		≤ 2	174(47)
		(2–5)	59(15.9)
		(5–8)	39(10.5)
		(8–12)	44(11.9)
		(12–18)	54(14.6)
Result of a consanguine marriage		Yes	223 (60.3)
		No	147 (39.7)
Type of relation		First-degree relation	150 (67.3)
		Second-degree relation	73 (32.7)
Cause of End-stage liver disease		Biliary atresia	135 (36.5)
		Progressive familial intrahepatic cholestasis	75 (20.3)
		Cryptogenic	38 (10.3)
		Tyrosinemia	21 (5.7)
		Biliary cirrhosis	20 (5.4)
		Wilson's disease	21 (5.7)
		Autoimmune hepatitis	16 (4.3)
		Paucity of intrahepatic bile ducts	8 (2.2)
		Primary sclerosing cholangitis	7(1.9)
		Glycogen storage diseases	5 (1.4)
		Alagille syndrome	5 (1.4)
		Neonatal hepatitis	6 (1.6)
		Caroli disease	2 (0.5)
		Cystic fibrosis	2 (0.5)
		Metabolic bile acid dysfunction	1 (0.3)
		Choledochal cyst	1 (0.3)
		Others	7 (1.9)
Clinical manifestations		Abdominal pain and ascites	62 (16.7)
		Jaundice	260 (79.5)
		Pruritus	31 (8.4)
		Melena	26 (7)
		Others	20 (5.4)
Blood group		A +	71 (25.8)
		A-	10 (3.6)
		B +	63 (22.9)
		B-	7 (2.5)
		O +	95 (34.5)
		O-	7 (2.5)
		AB +	21 (7.6)
		AB-	1 (0.4)
First PELD Score		—	15.8 ± 11.1
Parental education	Father	Illiterate to primary	73 (19.8)
		Secondary to diploma	217 (58.7)
		University degree	79 (21.3)
	Mother	Illiterate to primary	88 (24)
		Secondary to diploma	181 (49.3)
		University degree	98 (26.7)
Parental occupation	Father	Governmental	66 (18)
		Non-governmental	293 (80.1)
		Unemployed/other	7 (1.9)
	Mother	Governmental	21 (5.8)
		Non-governmental	5 (1.4)
		Housewife	335 (92.5)
		Other	1 (0.3)

## Mortality and Liver Transplantation

 Detailed information regarding primary outcomes including mortality and liver transplant among participants is provided in Table 2.

**Table 2:** Detailed Information on Mortality and Liver Transplant Among the Participants

**Variables**		**No. (%)**
Primary outcome	Alive	291 (78.6)
	Dead	79 (21.4)
Place of death	Hospital	66 (83.5)
	Home	11 (13.9)
	Clinic	1 (1.3)
	Unknown	1 (1.3)
Cause of death	Unknown	29 (36.7)
	Infection	10 (12.7)
	Massive gastrointestinal bleeding	8 (10.1)
	Multi-organ failure	7 (8.9)
	Hepatic encephalopathy	5 (6.3)
	Liver failure	4 (5.1)
	Rejection after liver transplant	4 (5.1)
	Heart failure	3 (3.8)
	COVID-19	2(2.5)
	Acute respiratory distress syndrome	2 (2.5)
	Disseminated intravascular coagulation	2 (2.5)
	Secondary bacterial peritonitis	2 (2.5)
	Hepatocellular carcinoma	1 (1.3)
Liver transplantation		92 (24.9)
Re-transplantation		5 (1.4)
Reason for re-transplant	Liver necrosis	2 (0.5)
	Rejection	3 (0.8)
Donor age	23 ± 4.2	-
Donor gender	Male	49 (54.4)
	Female	41 (45.6)
Type of Organ	Living donor	48 (52.2)
	Deceased donor	44 (47.8)
Transplant outcome	Alive	69 (75)
	Dead	23 (25)
